# *JBioWH*: an open-source Java framework for bioinformatics data integration

**DOI:** 10.1093/database/bat051

**Published:** 2013-07-11

**Authors:** Roberto Vera, Yasset Perez-Riverol, Sonia Perez, Balázs Ligeti, Attila Kertész-Farkas, Sándor Pongor

**Affiliations:** ^1^Department of Physics, Polytechnic University Jose E. Echeverria (ISPJAE), Havana, Cuba, ^2^Departament of Protein Structure and Bioinformatics, International Centre for Genetics Engineering and Biotechnology (ICGEB), Trieste, Italy, ^3^Department of Proteomics, Center for Genetics Engineering and Biotechnology (CIGB), Havana, Cuba, ^4^Proteomic Services, EMBL Outstation, European Bioinformatics Institute, Wellcome Trust Genome Campus, Hinxton, Cambridge, UK, ^5^Department of Informatics, ISPJAE, Havana, Cuba and ^6^Faculty of Information Technology, Pázmány Péter Catholic University, Budapest, Hungary

## Abstract

The Java BioWareHouse (JBioWH) project is an open-source platform-independent programming framework that allows a user to build his/her own integrated database from the most popular data sources. JBioWH can be used for intensive querying of multiple data sources and the creation of streamlined task-specific data sets on local PCs. JBioWH is based on a MySQL relational database scheme and includes JAVA API parser functions for retrieving data from 20 public databases (e.g. NCBI, KEGG, etc.). It also includes a client desktop application for (non-programmer) users to query data. In addition, JBioWH can be tailored for use in specific circumstances, including the handling of massive queries for high-throughput analyses or CPU intensive calculations. The framework is provided with complete documentation and application examples and it can be downloaded from the Project Web site at http://code.google.com/p/jbiowh. A MySQL server is available for demonstration purposes at hydrax.icgeb.trieste.it:3307.

**Database URL:** http://code.google.com/p/jbiowh

## Introduction

Integrating heterogeneous bioinformatics data is perhaps one of the most challenging tasks in biomedical research today. The fast accumulation of data is only one side of the problem. In addition, the number of data types used by different user groups in life sciences is also rapidly increasing [for recent reviews see (1)]. Integration of heterogeneous data is also one of the oldest themes in computer sciences. A complete overview of the subject would be beyond the scope of this article, as virtually all current bioinformatics efforts contain some kind of data integration [for in-depth reviews see (2–5)]. Traditionally, biological database integration efforts are classified into three main classes: federated, mediated and warehouse-style integration. Federated integration, (sometimes termed portal, navigational or link integration) provides hyperlinks to join data; early examples include SRS (6) and Entrez (7). The Semantic Web and linked data are a more recent approach that uses the World Wide Web to create typed links between data from different resources [for biological applications see (8)]. With the federated approach, it is relatively easy to provide up to date information but extra care is required to maintain the links. On the other hand, mediated integration [also called view integration (9–11)] provides a unified query interface and collects the results from various data sources. DiscoveryLink (10), BioMediator (9), BioMoby (12) are good examples of this approach. Finally, warehouse databases integrate data sources in one place: examples of this approach include BioWarehouse (13), Biozon (14), Atlas (15), EnsMart (16) and IGD (17). This approach provides fast querying over joined data sets, but also requires continuous updating.

Conceptual integration of data is a common issue in all database integration efforts. For instance, the contents and meaning of the term ‘protein’ are different in Uniprot (18) and in Drugbank (19). In a warehouse-style system, the two data sets would be linked under one overall scheme. On the other hand, an integrated concept would require a new data type to be implemented followed by appropriate matching of underlying entities and attributes, including the resolution of conflicts. It is often argued that a single biological database built along these lines would be a poor solution (2). Simply put, building a single unified database is possible only through a series of compromises that would ultimately limit the interpretation of the diverse data underlying the individual databases.

Large central resources such as those of the NCBI, the EBI and KEGG make great efforts to integrate various databases and data sets to provide a fundamental and high-level service for individual biologists around the world. The integrated databases available on the Internet [e.g. Ensemble (20), BioMart (21) and KEGG (22)] are good examples of this trend. However, querying distributed data and the use of public Internet resources comes with certain inherent limitations. For instance, concerns about confidentiality often prevent commercial enterprises from using such public services. Some academic research projects, such as artificial intelligence studies may require many thousands of queries to be executed within one experiment, which tends to be beyond the capacity of most public World Wide Web resources. For instance, training a machine-learning algorithm via database querying may require the retrieval of gigabytes of data for each query, which makes the process slow and vulnerable to network failures. And, some projects may require the flexible assembly of tailored data sets, which may be too time-consuming if done on a case-by-case basis. Emerging technologies such as molecular diagnostics in personalized medicine will raise analysis needs far beyond their current levels and will challenge the abilities of currently available systems and databases. Finally, Internet bandwidth in many countries makes the use of centralized services difficult, even for teaching courses. For these and similar reasons there is constant interest in building and maintaining integrated databases locally. The improvements in storage capacity (and falling costs) and CPU performance lend strong support to this tendency.

One interesting direction that is likely to further increase the interest in local data integration is the fast development of specialized bioinformatics computing environments. Examples of these include Bioperl (23), Biopython (24), BioJava (25) and Gaggle (26). These projects are community-based and are built on widely available languages such as Perl, Python and Java. They are also complemented with bioinformatics programming tools that enable individual users to build complex workflows from these open-source components. We believe that these tools have good potential for building high-capacity data-mining systems on PCs. Nevertheless, at the moment there are surprisingly few examples of open-source data integration frameworks running on personal workstations (5). As an example, the Atlas data warehouse (15) included data from 14 databases [GeneBank (27), RefSeq (7), Uniprot (18), NCBI Taxonomy (28), Gene Ontology (29), BIND (30), HPRD (31), IntAct (32), DIP (33), MINT (34), LocusLink (35), Gene (28), Homologene (28) and OMIM (36)]. Atlas achieved data integration at two levels. At the first level, common data models were used for data of similar types, enforcing the relationships between data types. The second level of integration was achieved by a combination of APIs, ontology and specific software tools. The data types supported by Atlas included taxonomies, ontologies, genes, proteins, protein–protein interactions and diseases. Unfortunately, Atlas is not available anymore.

Another well-known example is BioWarehouse, an open-source toolkit for building bioinformatics database warehouses in MySQL and Oracle (13). BioWarehouse integrates databases into a common representational framework within a single database management system (DBMS) so that data can be queried in Structured Query Language (SQL). Currently BioWarehouse supports the following data types: chemical compounds, biochemical reactions, metabolic pathways, proteins, genes, nucleic acid sequences, features on protein and nucleic-acid sequences, organisms, organism taxonomies and controlled vocabularies. The BioWarehouse includes data from 10 databases [GeneBank (27), Uniprot (18), NCBI Taxonomy (28), Gene Ontology (29), BioCyc and MetaCyc (37), BioPad (38), CMR (39), Enzyme (40) and KEGG (22)].

Despite the development of these data integration approaches, a number of specific challenges still remain. The format of data continually changes owing to new standards or new methods of data collection. Moreover, new cross-references are being added to databases, meaning that biological data integration projects must make special efforts to update integrational schemas to include cross-references between data sets as well as to add new databases. Open-source community-based projects hold a promise in this respect, which is why we decided to develop a data integration framework for the Java environment. Java BioWareHouse (JBioWH) is an open-source framework aiming to reach users who need to query multiple public data sets in a flexible way using their PC or a local workstation. JBioWH is based on the free and platform-independent Java language to create a MySQL-based data warehouse that is built around a modular relational database scheme. The aim of JBioWH is to allow users to construct application-specific databases; in this article, we present a demo example of integrating 20 public databases that contain 13 biological data types. The data are linked by cross-references, meaning that complex SQL queries can be executed. The project is continually updated by introducing new biological databases, extra functionalities to the Java API and new features to the Desktop Client.

## JBioWH Framework Content

**Figure 1.**
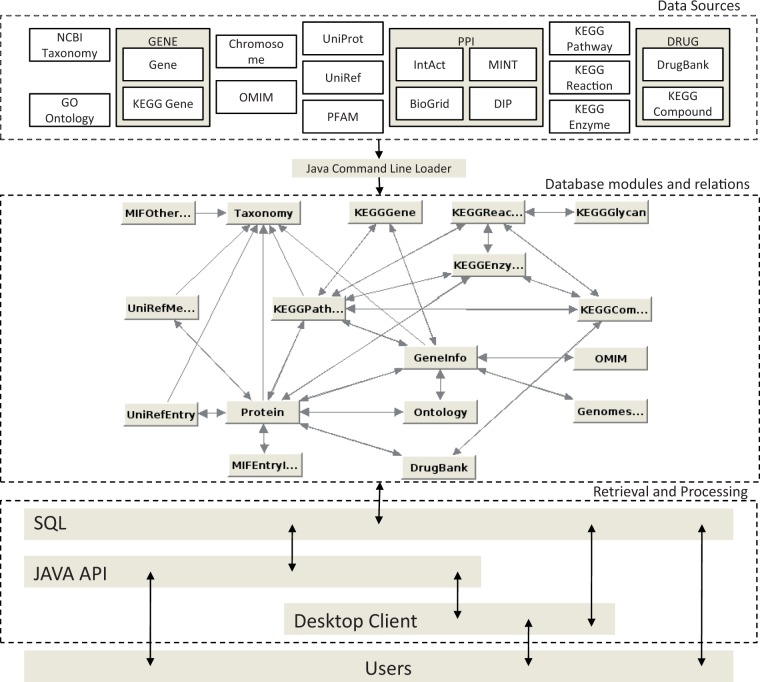
The JBioWH architecture.

The framework consists of three main parts: (i) the data sources, (ii) the relational schema and (iii) the Java API. The structure of the JBioWH can be seen on Figure 1. The following sections outline the JBioWH framework in detail.

### Data sources

JBioWH contains data retrieved from 20 public databases. Table 1 list these data sources along with their URL address. New databases will be added continually.

**Table 1.** Data sources included in JBioWH

Data Type	Data Source	URL	Data Format
Taxonomy	NCBI Taxonomy	ftp://ftp.ncbi.nih.gov/pub/taxonomy/taxdump.tar.gz	Delim. Text
Ontology	GO	ftp://ftp.geneontology.org/pub/go/godatabase/archive/latest-full/	OBO XML
Gene	Gene	ftp://ftp.ncbi.nlm.nih.gov/gene/DATA/	Delim. Text
Gene	KEGG Gene	http://www.bioinformatics.jp/en/keggftp.html	Text
Gene	GeneBank	ftp://ftp.ncbi.nih.gov/genbank	Text
Gene	RefSeq	ftp://ftp.ncbi.nih.gov/refseq/release/	Text
Chromosome	Genomes	ftp://ftp.ncbi.nih.gov/genomes/	Delim. Text
Protein	UniProt	ftp://ftp.uniprot.org/pub/databases/uniprot/current_release/knowledgebase/complete/	XML
Enzyme	KEGG Enzyme	http://www.bioinformatics.jp/en/keggftp.html	Text
PPI	IntAct	ftp://ftp.ebi.ac.uk/pub/databases/intact/current/psi25/pmidMIF25.zip	PSI 25 XML
PPI	MINT	ftp://mint.bio.uniroma2.it/pub/release/psi/current/psi25/pmids/	PSI 25 XML
PPI	DIP	http://dip.doe-mbi.ucla.edu/dip/	PSI 25 XML
PPI	BioGrid	http://thebiogrid.org/	PSI 25 XML
Prot. Cluster	UniRef	ftp://ftp.uniprot.org/pub/databases/uniprot/uniref/uniref100/	XML
Drug	DrugBank	http://www.drugbank.ca/system/downloads/current/drugbank.xml.zip	XML
Drug	KEGG Comp.	http://www.bioinformatics.jp/en/keggftp.html	Text
Pathway	KEGG Pathway	http://www.bioinformatics.jp/en/keggftp.html	Text
Reaction	KEGG Reaction	http://www.bioinformatics.jp/en/keggftp.html	Text
Disease	OMIM	http://www.omim.org/downloads	Text
Prot. Domain	PFAM	ftp://ftp.sanger.ac.uk/pub/databases/Pfam/releases/Pfam26.0/database_files/	SQL

The databases were accessed in October 2012.

### Relational schema

**Figure 2.**
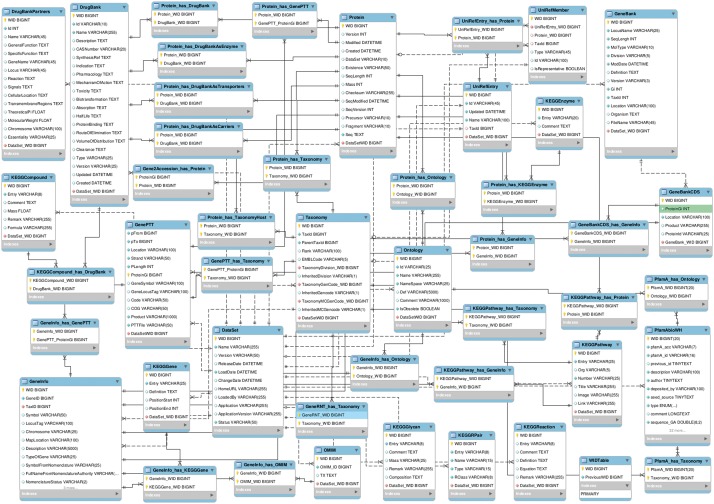
The JBioWH relational schema with the main tables and their relationships.

For the integrated database, we designed a relational database scheme in SQL. The relational scheme contains 363 tables including auxiliary tables for cross-references and for speeding up the ‘join’ operations. Figure 2 shows the main tables and their relationships in the relational schema.

### Java API packages

**Figure 3.**
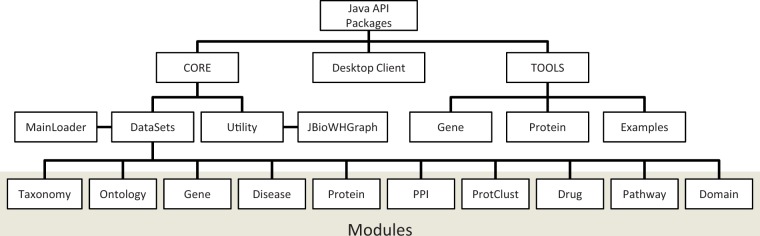
The structure of the Java API packages. The JAVA API contains (i) Core classes that define the data modules, (ii) the Desktop Client for non-programmer users and (iii) Tool package with command line programs and examples.

A Java API has been designed for maintaining the relational database and querying the data. Figure 3 shows the structure of the Java API packages. This API includes a Core package for the maintenance of JBioWH, a Desktop Client tool, and a Tool package that contains command line programs.

#### Core package

**Figure 4.**
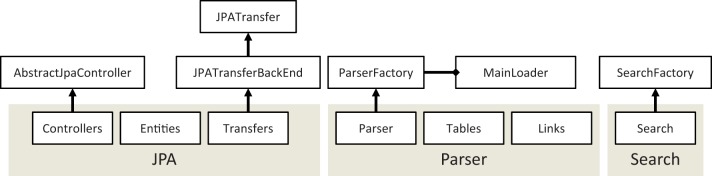
The content of the modules in the JAVA API. Each module defines (i) the JPA to manage the relational scheme in Java, (ii) Parser and Loader functions to load data from database sources to the JBioWH and (iii) Search classes to execute queries.

This package contains the main Java classes used for database maintenance. The imported biological data are grouped into 10 modules according to their biological type. The modules use the Java Persistence API (JPA) to manage the relational database in Java. The core classes use the Eclipse Link Library (version 2.3.2) to map object-oriented models onto MySQL tables in the back-end. The modules implement the abstract SearchFactory class to execute queries without using SQL. Each module implements loader and parser functions to connect to the source databases, retrieve data in flat file format, parse and put the data into the relational database. Modules can be loaded or unloaded independently from each other; hence, unnecessary data can be easily omitted to save storage space and to speed up query execution. The biological databases are often large and their syntax is often poorly defined. This problem frequently causes loader failures in the available integration frameworks, or worse, it can corrupt the biological data themselves. The loader functions of JBioWH were designed to preserve the biological data so that the loader process stops in case of errors in the database format or structure. The Java classes were designed according to the standard Java design patterns. Figure 4 shows the structure of modules in detail.

JBioWH also includes a global class called ‘JBioWHGraph’, which can be used to create complex graphs from queries. The graph created by this class is a set of vertices that are the biological objects (Taxonomy, Genes, Proteins, etc.) and a set of edges that are the relationship between them. The graph is created using a recursive algorithm following the JPA relationship between the entities. This approach permits the creation of directed or undirected graphs, including weighted edges, depending on the relationship between the vertices. In addition, the class provides a set of methods to retrieve some of the most common properties of the graph such as node connectivities, size and diameter of a graph, and it also allows operations like shortest path identification (for a complete description see http://code.google.com/p/jbiowh/wiki/JBioWHGraph).

The ‘JBioWHGraph’ class can be extended to create different kinds of graphs (see ‘Examples’ section below). This extension also offers a tool to create graphs using the biological objects as edges denoting a relationship between other objects that the protein is linked to. Currently three classes are available, the ‘TaxonomyGraph’ class, the ‘ProteinProteinGraph’ class and the ‘DrugPathwayGraph’ class. ‘TaxonomyGraph’ creates a directed graph with the hierarchical structure starting from a Taxonomy parent. This particular graph is useful in queries to be executed over an entire taxon such as a genus or family. ‘ProteinProteinGraph’ creates an undirected and weighted graph of protein–protein interactions. This class uses the Protein–Protein interaction data sets (such as MIF25) to create the edges between the proteins using the ‘MIFEntryInteraction’ class. The edges are weighted depending on how many times each interaction is reported by different data sets. The ‘DrugPathwayGraph’ class creates an undirected and weighted graph of drug pathways. This graph uses the proteins as edges creating the relationship between drugs and pathways. The edges are weighted depending on how many times each relationship (drug–pathway) is created by different proteins.

#### Desktop Client

**Figure 5.**
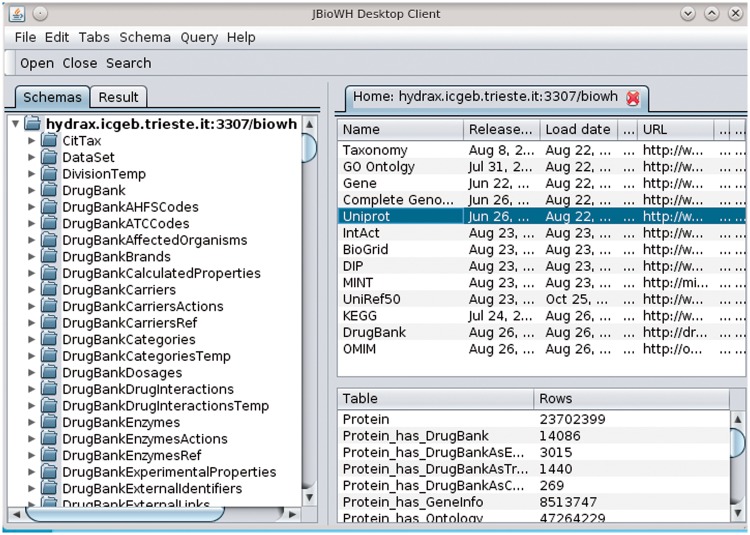
A screenshot of JBioWH Desktop Client. The left panel shows the relational schemes opened. The top right panel shows the list of the database inserted in the relational scheme, while on the bottom left panel one can see the tables in the selected database.

The JBioWH Desktop Client application has been developed for users who are not familiar with SQL script or the Java programming language. The client provides a graphical interface to access, manipulate and execute complex queries by simple mouse clicking from the integrated database (the desktop client is illustrated in Figure 5). A detailed user manual is available on the project’s Web site (http://code.google.com/p/jbiowh).

#### Tool package

The Tool package contains command line programs to execute quick queries.

## Implementation and Documentation

JBioWH can be used in any operating system that supports Oracle Java SE 7 or later, Apache Maven 2.2 or later (only for compilation) and MySQL Server 5.5 or later (only for the relational database). The Desktop Client can only be used with the Java virtual machine and the MySQL Java connector. The tables and the relational scheme have been implemented in a MySQL relational DBMS (version 5.5). The tables use the MYISAM engine and include the index creation syntax.

The system has been tested on Windows 7, Linux and in mixed environments where the MySQL DBMS was running on a Linux server and the Desktop Client was running on Windows. The project’s Web site at http://code.googlecode.com/p/jbiowh contains the complete documentation of the JBioWH including the (i) relational scheme diagrams for each module (this includes all tables, fields and links); (ii) a JavaDoc for the detailed description of Data Parsers and Desktop Client classes/methods; and (iii) a complete user manual for the JBioWH Desktop Client along with examples. A Google group called jbiowh-discuss was created for posting questions and ideas (https://groups.google.com/d/forum/jbiowh-discuss). A MySQL database server, designed for demonstration purposes, is available at hydrax.icgeb.trieste.it:3307.

JBioWH is under continuous development and includes the addition of further data sources, new functionalities, fixing bugs and adapting the format to changes in the data sources. Releases and updates will be provided via the project’s Web site. The JBioWH project provides a demo database hosted at one of our servers. This database is updated once in a month. If it is loaded with all the data, JBioWH requires ∼300 GB storage space (10 January 2013). On the other hand, JBioWH is modular, so only a small part of the data may be necessary for any given project. We also note that JBioWH does not contain the data themselves, and the end-user needs to get all the licenses and permissions to use the individual databases.

## Examples

**Figure 6.**
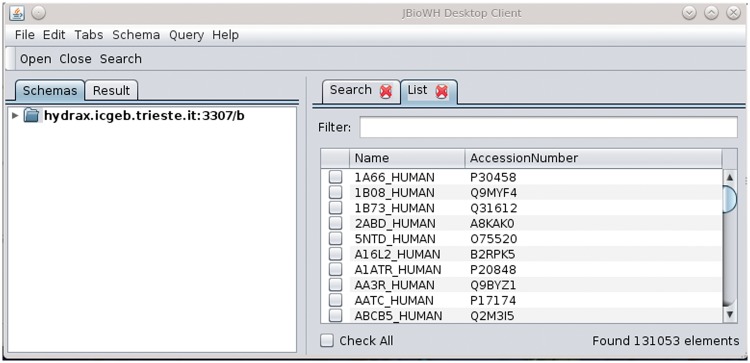
The solution of the second task in Table 2 using the JBioWH Desktop Client. The step-by-step guide on how to get this answer can be seen on the project Web site.

Examples of simple data retrieval (retrieving one protein sequence, or all human sequences) are shown in Table 2. The table shows two solutions for each problem, i.e. the SQL command for retrieving the data, and the Java code used in conjunction with the API. Figure 6 shows a solution for the second example (retrieving all human sequences) using the Desktop Client (a step-by-step tutorial is found in the project wiki pages (http://code.google.com/p/jbiowh/wiki/Ex1SearchTab).

**Table 2.** Two simple examples and their solutions using SQL language and the Java API code

Task	SQL solution	Java solution
Retrieve the protein sequence for the protein Q8DR59 from UniProt.	**select** p.seq **from Protein** p **inner join ProteinAccessionNumber** a **on a.Protein_WID = p.WID where a.AccessionNumber** = **'Q8DR59'**;	SearchProtein sProt = new SearchProtein(); **List** prots = sProt.search(“**Q8DR59**”, null); for(**Protein** p : (**List**<**Protein**>) prots) System.out.println(p.getSeq());
Retrieve the protein sequence of all human proteins	**select** p.seq **from Protein** p **inner join Protein_has_Taxonomy** pt **on pt.Protein_WID = p.WID inner join TaxonomySynonym** ts **on ts.Taxonomy_WID = pt.Taxonomy_ WID where ts.Synonym like** ‘**human'**;	SearchTaxonomy sTax = new SearchTaxonomy(); SearchProtein sProt = new SearchProtein(); **List** taxs = sTax.search(‘**human’**,null); List c = new ArrayList(); List o = new ArrayList(); c.add(taxs); o.add(“**IN**”); **JPLConstrains** constrain = new **JPLConstrains**(c,o,null); **List** prots = sProt.search(“”, constrain); for(**Protein** p : (**List**<**Protein**>) prots) System.out.println(p.getSeq());

More complex questions can be solved using the JBioWH API. These kinds of questions can not be solved directly using SQL language (neither the Desktop Client).

The JBioWH API can be used to answer queries that cannot be easily handled by the SQL language. Recursive queries such as finding nearest neighbors in terms of metabolism, taxonomy or chromosomal locations are typical examples of this kind of a problem. For instance, we want to find out if there are antibiotics that target a certain chromosomal region. As an example we take the ±10 genes the chromosomal neighborhood of the gene ‘spr0328’ of *Streptococcus pneumoniae* R6, which encodes the protein Endo-alpha N-acetylgalactosaminidase (Q8DR60). As the answer, JBioWH will find gene ‘spr0329’ (GeneId: 934791). This gene encodes protein ‘Q8DR59’, a penicillin-binding protein that is the target of Oxacillin, Hetacillin, Nafcillin, Ampicillin, Cefalotin, Azidocillin, Cefotaxime, Cefoxitin and Cephalexin. To answer this question one needs to retrieve gene and chromosomal position information from the Gene and Genome databases, respectively, to identify the corresponding proteins in the UniProt database. Subsequently, JBioWH retrieves antibiotic information for these proteins from DrugBank. The total execution time for this operation is 10 s. The complete description and the source code are available at https://code.google.com/p/jbiowh/wiki/Example7.

We can extend the scope of this question for an entire taxonomic subgroup. The question now is whether the orthologs of gene ‘spr0328’ in a certain taxonomic group have chromosomal neighbors that encode for antibiotic targets. We will use the ±10 genes neighborhoods in two genera, *Streptococcus* and *Burkholderia*. To answer this question we have to retrieve the taxonomic groups first. For this purpose *JBioWH* uses graph structures that can be created by extending the ‘BioWHGraph’ class in Java. For instance, the ‘TaxonomyGraph’ class represents the hierarchical structure of a Taxonomy family. Table 3 shows the data of three example taxonomies that can be created by a code shown in https://code.google.com/p/jbiowh/wiki/Example5.

**Table 3.** This table shows the use of the TaxonomyGraph class to create the hierarchical structure of a Taxonomy family

Family	Tax Id	Graph		Time (s)
		Vertex	Edges	
Bacteria	2	283 371	283 370	121
*Burkholderia*	32 008	3790	3789	4
*S. pneumoniae*	1313	303	302	3

To answer the question, we first want to get the orthologs of spr0328 from all *Streptococci*, located within a 10-gene neighborhood spr0328 and then locate the antibiotic target genes. For the location of the orthologs JBioWH uses UniRef clusters as an approximation [the COG (41) and the eggNOG (42) databases will be added in the near future]. In *Streptococcus* JBioWH finds two genes encoding antibiotic target proteins, gene 934791 of *S. pneumoniae* R6, which was already found in our first example, and gene 930269 *S. pneumoniae* TIGR4. The total execution time for this query was 15 s. The complete description and the source code are available in https://code.google.com/p/jbiowh/wiki/Example8.

Now we further generalize the query: Given a taxonomic group find all chromosomal regions (say maximum 5000 bp in length) that harbor at least two genes encoding antibiotic targets. Again, we will use the genera *Streptococcus* and *Burkholderia* as the examples. JBioWH will use ‘TaxonomyGraph’ class to retrieve the genus members. The NCBI PTT table of the genomes will be used for a step-by-step search. Genes that encode an antibiotic target will be identified through links to Uniprot, and from Uniprot to Drugbank. The results in Table 4 show that one gene-pair in *S. pneumoniae* TIGR4, and two gene-pairs in *Burkholderia xenovorans* LB400 are retrieved. The execution time for *Streptococcus* is one 112 s and for the *Burkholderia* 249 s. The complete description and the source code are available at https://code.google.com/p/jbiowh/wiki/Example6.

**Table 4.** This table shows the genes encoding for drug’s target protein that are in the same chromosome at a distance less than a specific number of pair bases

Family	Genes	Found gene ID	Specie	Time(s)
*S. pneumoniae*	41 576	930805-930802	*S. pneumoniae* TIGR4	112
*Burkholderia*	224 568	4010698-4010703	*B. xenovorans* LB400	249
		4010703-4010704		

Finally we show examples related to drugs that act on similar targets. In the database, the drugs are linked to proteins, and proteins are members of a network of metabolic pathways. In this system, two drugs can be (i) target neighbors, if they act on the same protein (ii) pathway neighbors, if they act on proteins that belong to the same metabolic pathway or (iii) distant neighbors, if they act on different pathways, and in the latter case it is important to know, in addition, how far apart in the metabolic network the two drugs are because distant relationships can be biologically meaningless. Questions related to (i) and (ii) can be answered by SQL queries but they need multiple joints, which makes the search time-consuming, especially in the case of (ii). Questions of type (iii), however, involve a prohibitively large number of multiple SQL join operations. JBioWH can handle these complex queries because of the graph structures implemented using the ‘DrugPathwayGraph’ class.

‘Hetacillin’ (DrugBank id: DB00739) is a beta-lactam that does not have intrinsic antibacterial activity, but is converted in the human body to Ampicillin, which is active against a variety of organisms. In DrugBank, Hetacillin is reported to act only on ‘Penicillin-binding protein 1A’ (UniProt Id: PBPA_STRR6) and ‘Penicillin-binding protein 2B’ (UniProt Id: PBP2_STRR6) both of which are parts of the ‘Peptidoglycan biosynthesis’ (KEGG: spr00550) of ‘*S. pneumoniae* R6’ (TaxId: 171101). Other links of Hetacillin are not reported even though its metabolite Ampicillin is well-known to act on various organisms. If we use a simple SQL query, only the links to ‘*S. pneumoniae* R6’ will be found. However, the ‘DrugPathwayGraph’ class can be used to find all target neighbors, pathway neighbors and also distant neighbors of the ‘Hetacillin’. The answer provided by JBioWH is that (i) the drug has 19 target neighbors that would act on the same protein target, (ii) has 38 pathway neighbors and (iii) the drug has 35 nearest distant neighbors that are identified as antibiotics. The execution time was 300–400 s.

In addition, the drug–pathway graph can be useful for identifying antibiotic drugs that target the same pathway in other organisms. For instance, ‘Ceftazidime’ (DrugBank Id: DB00438) and ‘Cyclacillin’ (DrugBank Id: DB01000) are antibiotic drugs that target the same pathway as Hettacillin, but in three different organisms, ‘*S. pneumoniae* R6’ (TaxId: 171101), ‘*Escherichia coli* str. K-12 substr. MG1655’ (Taxid: 511145) and ‘*Clostridium perfringens* str. 13’ (Taxid: 195102). This answer can be obtained using the ‘DrugPathwayGraph’ class as described in https://code.google.com/p/jbiowh/wiki/Example10.

Further examples can be seen on the project’s Web site http://code.google.com/p/jbiowh/wiki/Examples.

We point out that graph-based queries cannot be easily answered by SQL-based systems such as relational databases that do not have graph extensions, and these queries are practically impossible to answer using the traditional central resources or federated databases such as Biomart (11, 21). Although the data sources involved are well-known and sufficiently cross-referenced, it would require multiple visits from one database to another, which would make the process too time-consuming and complex for human operators, and also too vulnerable to network failure. On the other hand, such complex questions may arise in data mining projects where the queries need to be answered many times within a loop.

## Discussion and conclusions

The JBioWH framework provides an open-source Java API for integrating biological data from various public databases in a data warehouse manner. The aim of JBioWH is to allow users to construct application-specific databases; in this article, we present a demo example of integrating 18 data sources. The integrated database is hosted on a local computer and so it can be used for data-intensive calculations. This feature is especially important for queries that are not, or not easily, accommodated on central data resources. We note also that this feature could be important in environments with slow, or limited, Internet access. The relational schema of JBioWH is defined in a MySQL DBMS, and contains Java classes and parsers that load the modules of the JBioWH with data from public databases. Finally, JBioWH includes an API interface for programmatic access and a Desktop Client that lets users easily manipulate and query data via a graphical interface.

Future work will aim to integrate further biological data sources and the implementation of other DBMS such as PostgreSQL and Oracle. We continue developing the Desktop Client by extending it with new functionalities. Future releases will appear at the project’s Web site. Finally, we encourage users to send us their recommendations and requests via the jbiowh-discuss Google Group.
